# A Glycine-Rich RNA-Binding Protein, CsGR-RBP3, Is Involved in Defense Responses Against Cold Stress in Harvested Cucumber (*Cucumis sativus* L.) Fruit

**DOI:** 10.3389/fpls.2018.00540

**Published:** 2018-04-23

**Authors:** Bin Wang, Guang Wang, Fei Shen, Shijiang Zhu

**Affiliations:** Guangdong Provincial Key Laboratory of Postharvest Science of Fruits and Vegetables, Key Laboratory of Biology and Genetic Improvement of Horticultural Crops-South China, Ministry of Agriculture, College of Horticulture, South China Agricultural University, Guangzhou, China

**Keywords:** cucumber, harvested fruit, chilling sensitive, GR-RBP proteins, antioxidant system, defense response

## Abstract

Plant glycine-rich RNA-binding proteins (GR-RBPs) have been shown to play important roles in response to abiotic stresses in actively proliferating organs such as young plants, root tips, and flowers, but their roles in chilling responses of harvested fruit remains largely unknown. Here, we investigated the role of *CsGR-RBP3* in the chilling response of cucumber fruit. Pre-storage cold acclimation at 10°C (PsCA) for 3 days significantly enhanced chilling tolerance of cucumber fruit compared with the control fruit that were stored at 5°C. In the control fruit, only one of the six cucumber *CsGR-RBP* genes, *CsGR-RBP2*, was enhanced whereas the other five, i.e., *CsGR-RBP3, CsGR-RBP4, CsGR-RBP5, CsGR-RBP-blt801*, and *CsGR-RBP-RZ1A* were not. However, in the fruit exposed to PsCA before storage at 5°C, *CsGR-RBP2* transcript levels were not obviously different from those in the controls, whereas the other five were highly upregulated, with *CsGR-RBP3* the most significantly induced. Treatment with endogenous ABA and NO biosynthesis inhibitors, tungstate and L-nitro-arginine methyl ester, respectively, prior to PsCA treatment, clearly downregulated *CsGR-RBP3* expression and significantly aggravated chilling injury. These results suggest a strong connection between *CsGR-RBP3* expression and chilling tolerance in cucumber fruit. Transient expression in tobacco suggests CsGR-RBP3 was located in the mitochondria, implying a role for CsGR-RBP3 in maintaining mitochondria-related functions under low temperature. *Arabidopsis* lines overexpressing *CsGR-RBP3* displayed faster growth at 23°C, lower electrolyte leakage and higher *F*_v_/*F*_m_ ratio at 0°C, and higher survival rate at -20°C, than wild-type plants. Under cold stress conditions, *Arabidopsis* plants overexpressing *CsGR-RBP3* displayed lower reactive oxygen species levels, and higher catalase and superoxide dismutase expression and activities, compared with the wild-type plants. In addition, overexpression of *CsGR-RBP3* significantly upregulated nine *Arabidopsis* genes involved in defense responses to various stresses, including chilling. These results strongly suggest *CsGR-RBP3* plays a positive role in defense against chilling stress.

## Introduction

The plant glycine-rich proteins (GRPs) superfamily is characterized by the presence of semi-repetitive glycine-rich motifs (Sachetto-Martins et al., 2000; Mangeon et al., 2010). Currently, this superfamily is divided into five classes based on the arrangement of the glycine-rich domains (I–III and V) or on the presence of additional motifs or domains (IV), such as an RNA recognition motif (RRM), and zinc finger or cold-shock domains (Mangeon et al., 2010; Ortega-Amaro et al., 2015). In plants, members of the GRP family are involved in many cellular processes, such as pollen hydration and competition (Mayfield and Preuss, 2000), protoxylem growth (Ryser et al., 1997), cell elongation (Mangeon et al., 2009), root size determination (Amanda et al., 2016), seed germination (Rodríguez-Hernández et al., 2014), flowering (Streitner et al., 2008), and circadian rhythms (Schmal et al., 2013). Moreover, they are involved in responses to various abiotic stresses, including cold (Kim et al., 2005, 2010a,b; Peng et al., 2012), freezing (Shinozuka et al., 2006), dehydration (Wang et al., 2009), salt (Aneeta et al., 2002; Peng et al., 2012; Tan et al., 2014; Ortega-Amaro et al., 2015), drought (Wang et al., 2011;Yang et al., 2014), wounding (Gramegna et al., 2016), aluminum stress (Amanda et al., 2016), oxidative stress (Schmidt et al., 2010), and pathogenic infection (Xu et al., 2014; Kim et al., 2015). Plant responses to adverse environmental conditions are the result of regulation of gene expression that can occur at transcriptional and post-transcriptional levels. At post-transcriptional levels, the regulation is mainly achieved by RNA-binding proteins (RBPs) that contain one or more RNA-recognition motifs (RRMs) on their N-terminal half, plus a variety of auxiliary motifs on their C-terminal half (Kim J.Y. et al., 2007). Glycine-rich RNA-binding proteins (GR-RBPs) containing RRMs at the N-terminus, and a glycine-rich region at the C-terminus (Horvath and Olson, 1998; Bocca et al., 2005; Mangeon et al., 2010), have been reported to play important roles in post-transcriptional regulation of gene expression in plants under environmental stresses (Staiger et al., 2003; Nomata et al., 2004; Kim et al., 2008a, 2012; Streitner et al., 2010).

Although few plant *GR-RBPs* have so far been characterized, their roles in cold responses have attracted much attention. Some studies showed that *GR-RBPs* are highly upregulated under cold stress conditions, and their overexpression enhances chilling or freezing tolerance. Examples include *PpGR-RBP* from the moss *Physcomitrella patens* (Nomata et al., 2004), *AtRZ-1a* from *Arabidopsis* (Kim et al., 2005), *BnGR-RBP1* from *Brassica napus* (Kim et al., 2012), the three *GR-RBP2s* from *Camelina sativa* (Kwak et al., 2013), and *GR-RBP7* from *C. sativa* (Kwak et al., 2016). However, other studies showed that overexpression of these genes did not confer chilling or freezing tolerance, although *GR-RBP*s were significantly upregulated under cold stress. For example, in *Arabidopsis*, expression of *AtRZ-1b* and *AtRZ-1c* were markedly enhanced by cold stress, whereas *AtRZ-1b* and *AtRZ-1c* overexpression did not increase seed germination or seedling growth under cold stress conditions (Kim et al., 2010a). The transcript level of *GR-RBP4* increased markedly with cold stress, but *GR-RBP4* overexpression did not obviously enhance cold or freezing tolerance in *Arabidopsis* (Kwak et al., 2005).

Although some plant *GRP* genes are ubiquitously expressed (Kim et al., 2005; Kwak et al., 2016), *GRP* genes generally exhibit developmental and tissue-specific expression patterns (Ortega-Amaro et al., 2015). For example, *MhGR-RBP1* is abundantly expressed in young leaves but weakly expressed in roots and shoots (Wang et al., 2012). In stems and leaves of petunia plants, the levels of *PtGRP1* expression declined with developmental age of the tissue (Condit, 1993). *Arabidopsis GR-RBP4* is highly expressed in actively proliferating organs such as young plants, root tips and flowers, but weakly expressed in mature leaves and stems (Kwak et al., 2005). Tomato *Tmf-5* is preferentially expressed in immature fruit and is turned off during the ripening process (Santino et al., 1997). So far, most of the studies on *GRPs* have been carried out using vegetative parts, and a role in fruit, especially harvested fruit, is largely unexplored.

Cold acclimation refers to a process by which plants develop cold tolerance after an initial exposure to a critical temperature (Thomashow, 1999; Wang and Zhu, 2017). Past studies have shown that cold acclimation can effectively reduce chilling injury in harvested fruit (Chaudhary et al., 2014; Kashash et al., 2016; Li et al., 2017; Wang and Zhu, 2017; Zhang et al., 2017; Wang et al., 2018). Chilling tolerance induced by cold acclimation in harvested fruit is related to various physiological and molecular changes, such as upregulated *CBF* gene in mango fruit (Zhang et al., 2017), enhanced antioxidant activity in kiwifruit (Yang et al., 2013) and pomegranate fruit (Kashash et al., 2016), and differentially and orderly activated reactive oxygen species (ROS) scavengers in harvested cucumber fruit (Wang and Zhu, 2017). However, whether GR-RBPs are involved in cold acclimation of harvested fruit remains to be investigated.

Low temperature is an effective way to maintain quality of fruit during postharvest storage but it may cause chilling injury, especially in chilling-sensitive species. Chilling injury usually produces fruit with surface pitting, browning, or decay, which often severely affects quality and shortens storage life. Cucumber fruit is chilling-sensitive and exhibits symptoms of chilling injury when stored at temperatures below 7–10°C. However, our recent research demonstrated that cold acclimation initiated comprehensive defense responses in cucumber fruit, and among the many differentially accumulated proteins were two CsGR-RBPs (Wang et al., 2018). In the present study, physiological, molecular, and genetic approaches were used to investigate the link between *CsGR-RBP3* expression and chilling tolerance, to deepen our understanding of the roles of GR-RBP proteins in defense responses of plants to cold stress.

## Materials and Methods

### Treatment and Storage Conditions

Cucumber (*Cucumis sativus* L. cv Huaqing) fruit were harvested at commercial maturity from a farm in Yinan County, Shandong Province, China, and then immediately transported to the laboratory within 24 h. Cucumber fruit of uniform shape, weight, and maturity as well as freedom from visual defects, were selected. During 2013 to 2016, two experiments were conducted and they were repeated at least two times.

Experiment A was conducted to evaluate the effects of prestorage cold acclimation (PsCA) at 10°C on chilling tolerance and *CsGR-RBPs* gene expression in harvested cucumber fruit. The two treatments were fruit stored at 5°C (control) for 12 days, and PsCA for 3 days followed by storage at 5°C for 9 days.

Experiment B was carried out to investigate the effects of inhibition of endogenous ABA and NO on PsCA-induced chilling tolerance and *CsGR-RBP3* expression. There were four treatments, i.e., control, PsCA, TS+PsCA, and L-NAME+PsCA. TS (tungstate) is an ABA biosynthesis inhibitor. L-NAME (L-nitro-arginine methyl ester) is a nitric oxide biosynthesis inhibitor. For application of the combination treatments, the fruit were first sprayed with TS (50 μM) or L-NAME (100 μM) and incubated at 20°C for 6 h before cold acclimation (PsCA) was applied.

All treated fruit were wrapped with perforated polyethylene film (0.03 mm in thickness) before storage. Each treatment was applied to three replications of 60 fruit. Of the 60 fruit, 30 were labeled for observation of chilling injury severity, and the remainder were used for destructive sampling. Peel tissues were collected at 2 days intervals. The three tissue samples from the same treatment were then pooled, ground to powder in liquid nitrogen, and stored at -80°C.

### Chilling Injury, Disease, and Electrical Conductivity Measurements

Treated fruit were sorted into five categories according to the chilling severity. Chilling injury indices (CII) and secondary disease indices (SDI) of cucumber fruit were evaluated using the 0–4 scale as described previously (Wang et al., 2018). Chilling injury development was observed during storage at 5°C, and secondary disease development was observed at ambient temperature (20°C) following 12 days of cold storage.

Twenty disks of cucumber peel tissues, or *Arabidopsis* leaves, were excised with a stainless steel cork borer (5 mm in diameter), washed three times with double distilled water, put into 25 mL of double distilled water, and then placed at ambient temperature for 2 h before electric conductivity of the solution (C1) was determined using a conductance bridge (DDS-307, Leici Electron Instrument Factory, Shanghai). The peel or leaf disks were then incubated for 10 min in a boiling water bath, cooled to 25°C, and electrical conductivity determined again (C2). Electrolyte leakage (EL, %) = (C2 - C1) × 100/C2 (Liu et al., 2016).

### Chlorophyll Fluorescence Measurements

Chlorophyll fluorescence is an indirect indicator of the physiological status of chlorophyll-containing tissues. It has been widely used to reflect chilling tolerance in plants (Xia et al., 2009; Wang et al., 2016). Here, chlorophyll fluorescence was measured with a portable chlorophyll fluorometer (IMAG-K7, Walz, Germany) as described previously (Wang et al., 2018). The cucumber fruit, or *Arabidopsis* plants, were dark-adapted for 30 min prior to measuring PSII quantum yield (*F*_v_/*F*_m_) on cucumber peels and *Arabidopsis* leaves.

### Proteomic Analysis

Total proteins from cucumber peel tissues were extracted for two-dimensional electrophoresis (2-DE) following Wang et al. (2018), with 17 cm IPG strips (pH 3–10, Bio-Rad). Protein concentration was measured by the Bradford method with bovine serum albumin (BSA) as the standard (Bradford, 1976). At least three biological replicates were used for each treatment and at least one gel was run for each replicate. After staining with Coomassie blue, PDQuest 2-DE analysis software (Version 8.0, Bio-Rad) was used to analyze the 2-DE images (Wang et al., 2014). Automated detection and manual editing were carried out to obtain the highest gel matching. To account for quantitative variations, the data were normalized between samples using the total intensity of valid protein spots based on the corresponding gel. The normalized intensity of protein spots on three independent replicate 2-DE gels was averaged.

The spots that showed more than twofold differences between treatments were excised and subjected to MS analysis (4700 proteomics Analyzer, Applied Biosystems) (Wang et al., 2018). MS spectra were acquired by the positive ion reflector mode. The five most abundant precursor ions were selected for MS/MS scans. All acquired spectra were processed with Flex Analysis 3.3 software (Bruker). Database searching was performed using Mascot software 2.3.02 (Matrix Science, United Kingdom) against the Cucumber Genomic database^1^. The cut-off score for accepting individual MS/MS spectra considered to be significantly different was 61 (Jamesdaniel et al., 2012). Results with the highest scores were considered as relevant for each identified protein. The theoretical mass (*Mr*) and isoelectric point (*pI*) were determined using the online Expasy tool^2^.

### *CsGR-RBP3* Gene Isolation and Sequence Analysis

The Open Reading Frame (ORF) of *CsGR-RBP3* was obtained from the Cucumber Genomic database^1^. This sequence was verified by further cloning and sequencing from cucumber peel tissues. The specific primers (forward, F1; reverse, F2) used for PCR amplification are listed in Supplementary Table S2. Conditions for PCR amplification were: 35 cycles of 94°C for 0.5 min, 60°C for 0.5 min, 72°C for 1 min, then 72°C for 10 min.

Gene sequence data were analyzed using the programs provided by the NCBI database. Multiple alignments of amino acid sequences were analyzed using CLUSTALX (version 2.0) and DNAMAN (version 6.0) programs. A phylogenetic tree of GRPs between cucumber and six other plants was constructed using the UPGMA method in the MEGA5 program.

### Subcellular Localization of CsGR-RBP3 Protein

The coding sequence without the stop codon was amplified by PCR. The specific primers (F3 and F4) are listed in Supplementary Table S2. The PCR product was cloned into a transient expression vector (pCAMBIA 2300-GFP) between the *Kpn I* and *Spe I* sites. The digested pCAMBIA 2300-GFP fragment and *CsGR-RBP3* fragment were linked with T4-ligase (Invitrogen, United States). *CsGR-RBP3-GFP* was driven by a cauliflower mosaic virus (CaMV) 35S promoter. The fusion constructs and control vectors were electroporated into *Agrobacterium tumefaciens* strain GV3101 using Gene Pulser Xcell^TM^ Electroporation Systems (Bio-Rad, United States). Tobacco (*Nicotiana benthamiana*) leaves were used for subcellular localization assay using the infiltration method. GFP fluorescence was observed using a fluorescence microscope 574 (Zeiss Axioskop 2 Plus) (Ye et al., 2016).

### Over-Expression of *CsGR-RBP3* in *Arabidopsis*

For heterologous expression of the *CsGR-RBP3* gene, the coding sequence was transferred to pCAMBIA 2300 at the *Kpn I* and *Spe I* sites using T4 ligase, and fused with the 35S CaMV promoter. The construct pCAMBIA 2300-*CsGR-RBP3* was then electroporated into GV3101 and transformed into *Arabidopsis* using the floral dip method (Zhang et al., 2006). Seed were harvested and sown onto MS selection medium containing kanamycin (50 μg/mL) for identification of the transgenic plants using the method described previously (Zhang et al., 2016). Two independent *35S::CsGR-RBP3* lines were obtained and used for further analysis. Plants were grown in growth chambers with a photoperiod of 16 h (13,000 lux)/8 h, and the light/dark cycle at temperatures of 23/16°C, respectively. DNA and mRNA extracted from the T1, T2, and T3 plants were used as templates to perform PCR using *CsGR-RBP3* gene-specific primers (F1 and F2, see Supplementary Table S2). T3 homozygous seedlings of two transgenic lines were used for analysis.

### Phenotype Analysis of Transgenic *Arabidopsis* Plants

Phenotype analysis was assayed with the method described previously (Zhang et al., 2016). Seed were sterilized with 75% (v/v) ethanol solution for 1 min and with 2% (v/v) chlorine solution for 10 min, and then rinsed four to five times in sterile distilled water. The sterilized seed were sown on MS medium, and the plates were incubated at 4°C for 2 days in the dark before germination before being grown-on in a growth chamber at 23°C with 16/8 h light/dark photoperiod. Primary root lengths were measured at 14 days, and rosette leaf numbers were counted at 22 days after sowing. For evaluating leaf growth rate, 22-day-old plants were incubated for 1 week under normal (23°C) or chilling (0°C) temperature conditions.

### Chilling and Freezing Tolerance Tests of Transgenic *Arabidopsis* Plants

Twenty two-day-old *Arabidopsis* wild-type and transgenic plants were used for evaluating chilling and freezing tolerance. For chilling treatment, plants were subjected to chilling stress at 0°C for 6 days with a 16 h/8h light/dark regimen. *F*_v_/*F*_m_ and EL were measured at 2-day intervals. For freezing tolerance test, plants were subjected to a freezing shock at -20°C for 15 min, and then transferred to normal growth conditions. The surviving plants were counted 7 days after transfer.

### Measurements of ROS Accumulation and Antioxidant Enzyme Activities

Twenty two-day-old *Arabidopsis* plants subjected to cold stress at 0°C for 6 days with a 16/8 h light/dark regimen were used to measure reactive oxygen species (ROS) accumulation and anti-oxidant enzyme activities. For localization of hydrogen peroxide (H_2_O_2_) and superoxide radicals (O_2_^⋅-^), the excised leaves were incubated in a 1 mg mL^-1^ nitroblue tetrazolium (NBT) solution (pH 3.8) or in a 1 mg mL^-1^ diaminobenzidine (DAB) solution (Sigma, Germany) for 8 h in the dark, respectively (Xia et al., 2009; Tan et al., 2014). H_2_O_2_ and O_2_^⋅-^ concentrations were determined following the method described previously (Wang and Zhu, 2017). H_2_O_2_ concentration was assayed by monitoring the absorbance of the titanium-peroxide complex at 415 nm. For determination of O_2_^⋅-^ concentration, samples were reacted with 1 mL of hydroxylamine hydrochloride for 1 h, then 1 mL of p-aminobenzene sulfonic acid and 1 mL of α-naphthylamine were added, and the mixture kept at 25°C for 20 min. O_2_^⋅-^ concentration was determined at 530 nm and was calculated using NaNO_2_ as standard curve. The determined H_2_O_2_ and O_2_^⋅-^ concentrations were expressed on a fresh weight basis, as μmol g^-1^.

Superoxide dismutase (SOD) and catalase (CAT) activities were measured following Wang and Zhu (2017). SOD activity was assayed by measuring the reduction of nitroblue tetrazolium chloride at 560 nm. CAT activity was assayed by measuring the initial rate of H_2_O_2_ decomposition at 240 nm in a reaction with 10 mM H_2_O_2_. SOD and CAT activities were calculated and expressed on a fresh weight basis, as unit g^-1^.

### RNA Extraction and Gene Expression Analysis

Total RNA from cucumber peel tissues and from *Arabidopsis* plants were extracted using Trizol reagent (Invitrogen, United States) according to the manufacturer’s instructions (Liu et al., 2016; Zhang et al., 2016). Genomic DNA was digested by RNase-free DNaseI. The DNA-free total RNA was used to synthesize cDNA. The cDNA was synthesized using an iScript cDNA Synthesis Kit (Bio-Rad, United States) following the manufacturer’s instructions (Liu et al., 2017).

Quantitative real-time PCR was used for the analysis of gene transcript accumulation following the method described previously (Wang and Zhu, 2017). Expression values were normalized using cucumber actin (*CsActin*) or *Arabidopsis* actin *(AtActin)*. The relative expression levels of target genes were calculated using the formula (2^-ΔΔC_t_^) (Livak and Schmittgen, 2001). The specific primers (Supplementary Table S2) were designed according to cDNA sequences using the Primer-BLAST tool of the NCBI (National Center for Biotechnology Information) database.

### Experiment Design and Statistical Analysis

The experiments were completely randomized designs. Treatments were applied to three replications of 30 fruit for CII and SDI evaluations, of 60 seedlings for measurement of primary root length, of 30 plants for measurement of survival rate and rosette leaf number, and of 18 plants for determination of *F*_v_/*F*_m_ and REL. Data were analyzed by one-way analysis of variance (ANOVA). Statistically significant differences were assumed at *P* ≤ 0.05.

## Results

### PsCA Induces Chilling Tolerance in Cold-Stored Cucumber

**FIGURE 1:**
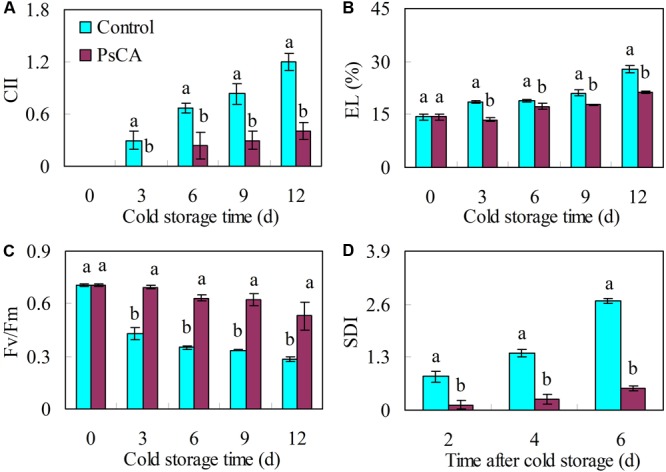
Effects of pre-storage cold acclimation (PsCA) on chilling tolerance in cold-stored cucumber. Chilling injury indices (CII) **(A)**, electrolyte leakage (EL) **(B)** and chlorophyll fluorescence (*F*_v_/*F*_m_) **(C)** were evaluated during storage at 5°C. Secondary disease indices (SDI) **(D)** were evaluated after the cucumbers were transferred to 20°C following 12 days of storage at 5°C. Fruit were first incubated at 10°C for 3 days and then stored at 5°C (PsCA) or were directly placed at 5°C (Control). Significant differences between the control and PsCA treatment are indicated by letters above each bar (*P* ≤ 0.05). Data are presented as means ± standard errors (±*SE*) (*n* = 3).

Compared with the control, PsCA significantly reduced CII and EL by 66.7% and 23.6%, respectively, at the end of cold storage (**Figures 1A,B**). PsCA treatment produced higher *F*_v_/*F*_m_ ratios than in the control during cold storage. After 12 days of storage, the *F*_v_/*F*_m_ ratios in the control decreased by 60.0%, while those of the PsCA-treated fruit decreased by 24.7% (**Figure 1C**). Chilling injury is generally followed by an increased tendency to decay when the temperature is raised (DeEll et al., 2000; Wang and Zhu, 2017). Therefore, SDI was determined at ambient temperature following 12 days of cold storage at 5°C. The results showed that the SDI in PsCA-treated cucumbers was 80.0% lower than in the control at 6 days in ambient temperature (20°C) (**Figure 1D**).

### Expression of *CsGR-RBP* Genes in Cucumber in Response to PsCA Treatment

To choose a representative from the *CsGR-RBP* gene family for studying the role of *CsGR-RBPs* in cold responses in harvested cucumber fruit, all the known *CsGR-RBP* genes found in the GenBank were monitored in terms of expression pattern. Among them, CsGR-RBP3 was previously identified by the proteomic approach in PsCA-treated cucumber fruit (Wang et al., 2018), and CsGR-RBP-blt801 was identified in cold-stored cucumber fruit exposed to PsCA plus cold storage (Supplementary Figure S1 and Supplementary Table S1).

**FIGURE 2:**
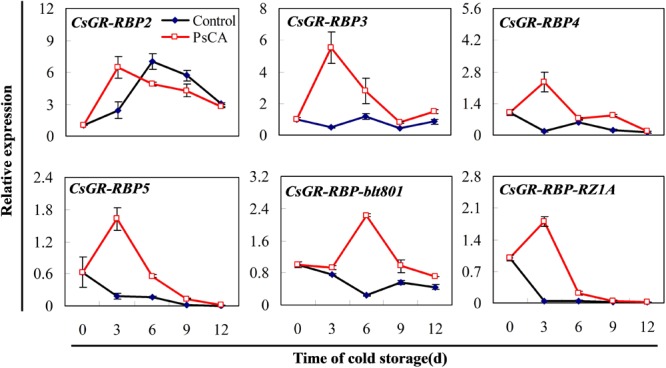
Effects of pre-storage cold acclimation (PsCA) on relative expression of *CsGR-RBP* genes in cold-stored cucumber. Fruit were first incubated at 10°C for 3 days and then stored at 5°C (PsCA) or were directly placed at 5°C (Control). The relative expression was evaluated by quantitative real-time PCR (qRT-PCR) using gene-specific primers (Supplementary Table S2) and the expression data were all normalized to 100% (1.0) at 0 day of the control. Gene names and the corresponding Genbank accession numbers are: *CsGR-RBP2* (XM_011656066.1), *CsGR-RBP3* (XM_004137389.2), *CsGR-RBP4* (XM_011651346.1), *CsGR-RBP5* (XM_004148797.2), *CsGR-RBP-blt801* (XM_011650146.1), and *CsGR-RBP-RZ1A* (XM_011660470.1). Data are presented as means ± *SE* (*n* = 3).

The expression pattern of the six genes can be divided into two categories (**Figure 2**). The first category consists of only *CsGR-RBP2*, whereas all the others fall into the second category. As is shown for the control, gene expression of *CsGR-RBP2* was highly upregulated under cold stress, whereas the other five genes were significantly downregulated, except for *CsGR-RBP3*, which showed little change during the 12 days in cold stress. For *CsGR-RBP2*, PsCA enhanced peak expression, but had little effect on the expression levels. On the other hand, all the genes in the second category were highly upregulated following PsCA treatment (**Figure 2**). Therefore, they could be considered as candidates for studying roles of *CsGR-RBPs* in cold responses. Among the five candidates, the transcript levels of *CsGR-RBP3* showed the highest PsCA/Control ratio at 3 days, the end point of the PsCA treatment, indicating *CsGR-RBP3* is the most highly upregulated transcript during PsCA. Therefore, *CsGR-RBP3* was selected for further investigation in this study.

### Expression of *CsGR-RBP3* in Relation to Chilling Tolerance

**FIGURE 3:**
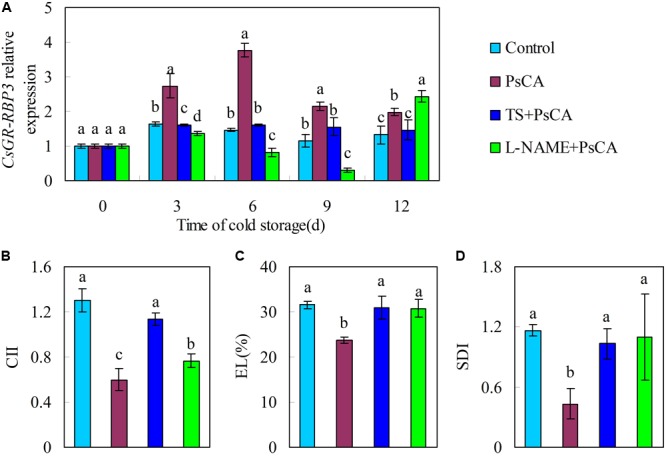
Effects of endogenous ABA and NO inhibitor application before PsCA treatment on *CsGR-RBP3* expression **(A)** and chilling severity **(B–D)** in cucumber fruit. **(A)** Relative expression of *CsGR-RBP3* was evaluated by qRT-PCR using gene-specific primers (Supplementary Table S2) and the expression data were normalized to 100% (1.0) at 0 day of the control. TS: tungstate, abscisic acid (ABA) biosynthesis inhibitor. L-NAME: L-nitro-arginine methyl ester, nitric oxide (NO) biosynthesis inhibitor. Fruit were first incubated at 10°C for 3 days and then stored at 5°C (PsCA) or were directly placed at 5°C (Control). For application of combination treatments, cucumbers were first sprayed with TS (50 μM) or L-NAME (100 μM), incubated for 6 h before exposure to cold acclimation. CII **(B)** and EL **(C)** were evaluated following 12 days of cold storage at 5°C. SDI **(D)** were evaluated at 2 days after the fruit were transferred to ambient temperature (20°C). Significant differences between treatments are indicated by letters above each bar (*P* ≤ 0.05). Data are presented as means ±*SE* (n = 3).

In order to confirm the relation between gene expression of *CsGR-RBP3* and chilling tolerance, tungstate (TS), an ABA biosynthesis inhibitor, and L-nitro-arginine methyl ester (L-NAME), a nitric oxide biosynthesis inhibitor, were applied to cucumber before exposure to cold acclimation. The combination treatments of TS+PsCA and L-NAME+PsCA significantly downregulated *CsGR-RBP3* expression compared with the PsCA treatment (**Figure 3A**). The two combination treatments significantly increased CII, EL, and SDI relative to PsCA alone (*P* ≤ 0.05) (**Figures 3B–D**). This shows that decrease in *CsGR-RBP3* gene expression was connected with reduced chilling tolerance in harvested cucumber.

### Sequence and Localization Analysis of *CsGR-RBP3*

**FIGURE 4:**
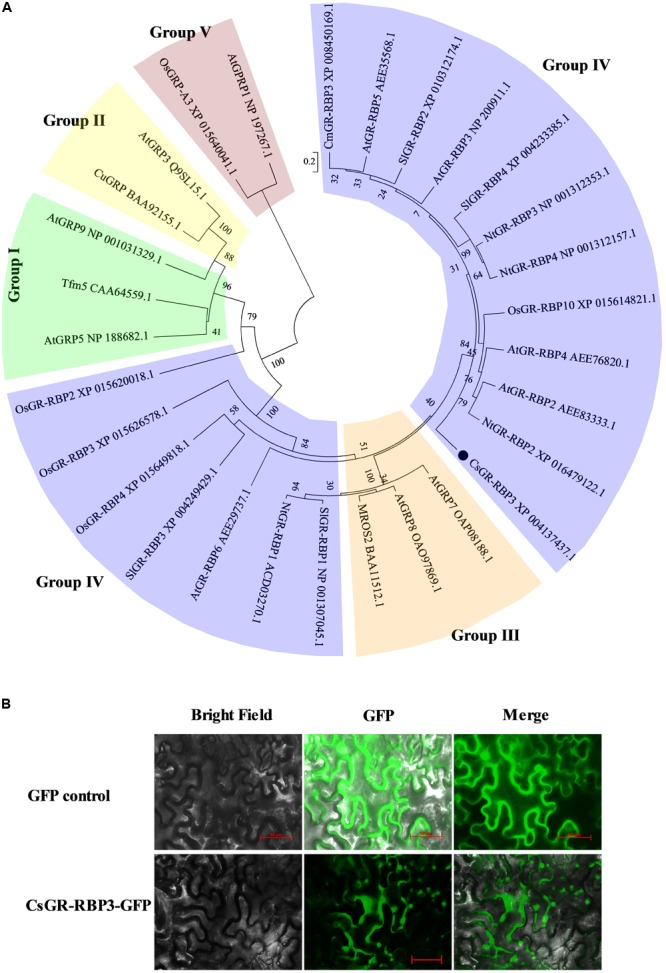
Phylogenetic analysis and subcellular localization of CsGR-RBP3. **(A)** Phylogenetic tree based on comparisons between CsGR-RBP3 protein sequence and GRPs from six other plant species. The GRPs are divided into five major groups. The phylogenetic tree was produced using MEGA5. Species names are abbreviated as follows: At, *Arabidopsis thaliana*; Cm, *Cucumis melo*; Cs, *Cucumis sativus*; Cu, *Citrus unshiu*; Nt, *Nicotiana tabacum;* Os, *Oryza sativa*; Sl, *Solanum lycopersicum*. The accession numbers are indicated. CsGR-RBP3 is indicated by the black circle. **(B)** Subcellular localization of CsGR-RBP3 in tobacco leaves. The coding sequence of *CsGR-RBP3* was cloned into a transient expression vector (pCAMBIA 2300-GFP) driven by the CaMV 35S promoter. The fusion constructs and control vector were electroporated into *Agrobacterium tumefaciens* strain GV3101, which were then infiltrated into tobacco (*Nicotiana benthamiana*) leaves. After 72 h of infiltration, GFP fluorescence was imaged using a fluorescence microscope. The length of red bars is 50 μm.

To further investigate the potential roles of *CsGR-RBP* genes in response to cold stress, the full-length cDNA of *CsGR-RBP3* was cloned from peel of cucumber fruit and sequenced. The Open Reading Frame (ORF) of *CsGR-RBP3* gene encompasses a length of 507 bp, with five exons separated by four introns (Supplementary Figure S2A). The encoded protein is basic with a predicted isoelectric point of pH 9.54 and MW of 18.24, and consists of 168 amino acids. It is characterized by an RRM conserved domain in sequence (Supplementary Figures S2B, S3) and 13 (GGX)_n_ repeats (Supplementary Figure S2C). Phylogenetic analysis showed that CsGR-RBP3 was classified into Group IV (**Figure 4A**). Alignment of the putative cucumber CsGR-RBP3 with 24 GR-RBPs from 6 other plants, i.e., *Arabidopsis thaliana, Oryza sativa, Cucumis melo, Citrus unshiu, N. tabacum*, and *Solanum lycopersicum*, showed identity between 15.1 and 48.2%. CsGR-RBP3 shared the highest identity with *CmGR-RBP3* from *Cucumis melo* (48.2%) (Supplementary Table S3).

A CsGR-RBP3-GFP fusion construct driven by the CaMV 35S promoter was used for the sub-cellular localization assay. Results show that the GFP control was observed in the nucleus and cytoplasm of tobacco cells, but CsGR-RBP3-GFP was visualized in the cytoplasm and in some organelles within the cytoplasm (**Figure 4B**). The predicted CsGR-RBP3 sub-cellular localization was mitochondrial using the Softberry website (Supplementary Table S4). This implies that CsGR-RBP3 could be a mitochondrial protein.

### Phenotypes of Transgenic *Arabidopsis* Plants Over-Expressing *CsGR-RBP3*

For heterologous expression in *Arabidopsis, CsGR-RBP3* was cloned into the *pCAMBIA 2300* expression vector (Supplementary Figure S4). After the recombinant vector was transformed into *Arabidopsis* plants, two kanamycin-resistant lines were generated (Supplementary Figure S5A). PCR analysis using DNA from T1 and T2 generations as templates confirmed that the *CsGR-RBP3* gene was successfully transformed into *Arabidopsis* plants (Supplementary Figures S5B,C). Semi-quantitative PCR, using cDNA from T3 generations as templates, confirmed that *CsGR-RBP3* was stably expressed in *Arabidopsis* (Supplementary Figure S5D).

**FIGURE 5:**
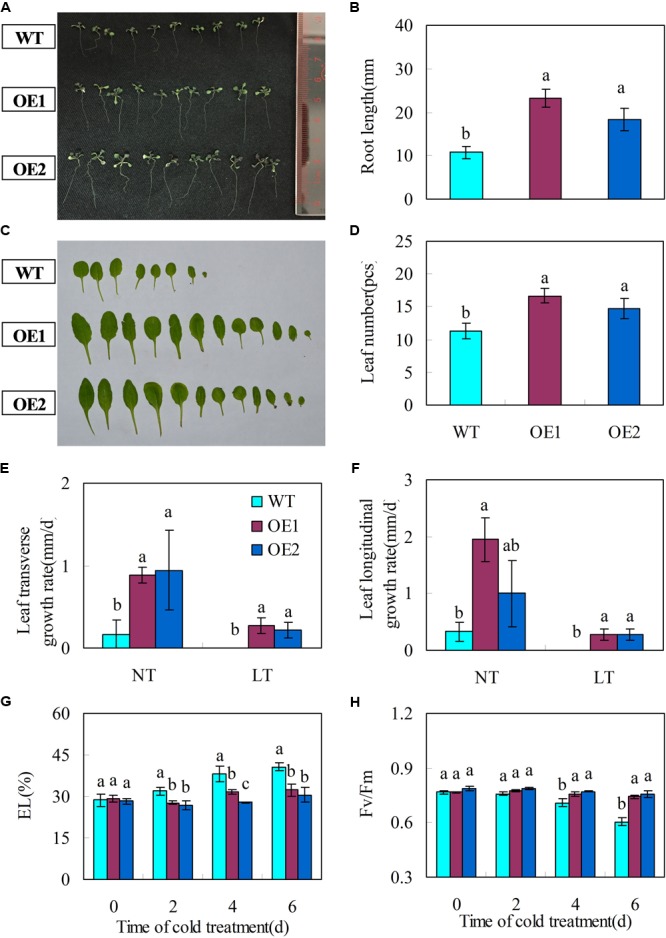
Transgenic *Arabidopsis* plants over-expressing *CsGR-RBP3* display fast growth and chilling tolerance. **(A)** Were taken and primary root lengths **(B)** were measured at 14 days after sowing. **(C)** Were taken and rosette leaf numbers **(D)** were counted at 22 days after sowing. The photograph shows a representative picture of repeated experiments. To determine leaf transverse **(E)** and longitudinal growth rates **(F)**, 22-day-old plants were subjected to chilling (0°C) temperature for 1 week. WT, wild-type; OE1 and OE2, two independently transgenic lines. NT, normal temperature (23°C); LT, low temperature. For evaluating electrolyte leakage (EL) **(G)** and chlorophyll fluorescence (*F*_v_/*F*_m_) **(H)**, 22-day-old plants of the wild type and *35S::CsGR-RBP3* transgenic plants (OE1 and OE2) were subjected to 0°C for 6 days. Significant differences between the control and PsCA treatment are indicated by letters above each bar (*P* ≤ 0.05). Data are presented as means ±*SE* (n = 10–30).

The transgenic plants had significantly longer primary root lengths (**Figures 5A,B**) and more rosette leaves than the wild-types (**Figures 5C,D**).

### *CsGR-RBP3* Confers Chilling and Freezing Tolerance in *Arabidopsis* Plants

**FIGURE 6:**
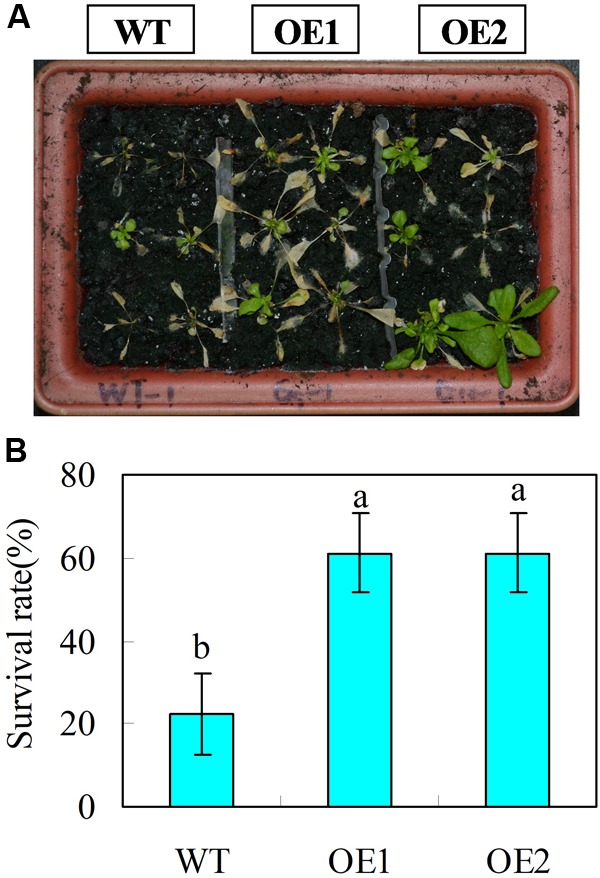
Transgenic *Arabidopsis* plants over-expressing *CsGR-RBP3* display freezing tolerance. 22-day-old plants were subjected to freezing shock at –20°C for 15 min, and then transferred to ambient growth conditions. **(A)** Were taken and survival rates **(B)** were evaluated at 7 days after transferring plants to ambient growth conditions. The photograph shows a representative picture of repeated experiments. Significant differences between the control and PsCA treatment are indicated by letters above each bar (*P* ≤ 0.05). Data are presented as means ±*SE* (*n* = 3).

Leaf growth rate was evaluated for 22-day-old wild type and *CsGR-RBP3-*over-expression lines. Under normal temperature, transgenic plants exhibited faster growth rate than those of wild-type plants, as reflected by transverse and longitudinal leaf growth rates (**Figures 5E,F**). However, under low temperature (0°C), no leaf expansion occurred on wild-type plants, whereas leaves on the transgenic lines continued to expand (**Figures 5E,F**). When 22-day-old plants were subjected to 0°C for 6 days, the transgenic plants showed lower EL but higher *F*_v_/*F*_m_ ratios than the wild-type plants (**Figures 5G,H**). Another set of 22-day-old plants were subjected to -20°C for 15 min. After the freeze-shock treatment, plants were transferred to ambient conditions and the survival rate was calculated following a 7 days period of recovery. The results showed that more than 60% of the over-expression lines recovered but only 20% of wild-type plants were able to survive (**Figures 6A,B**).

### *CsGR-RBP3* Enhances Antioxidant Capacity in Transgenic *Arabidopsis* Plants

**FIGURE 7:**
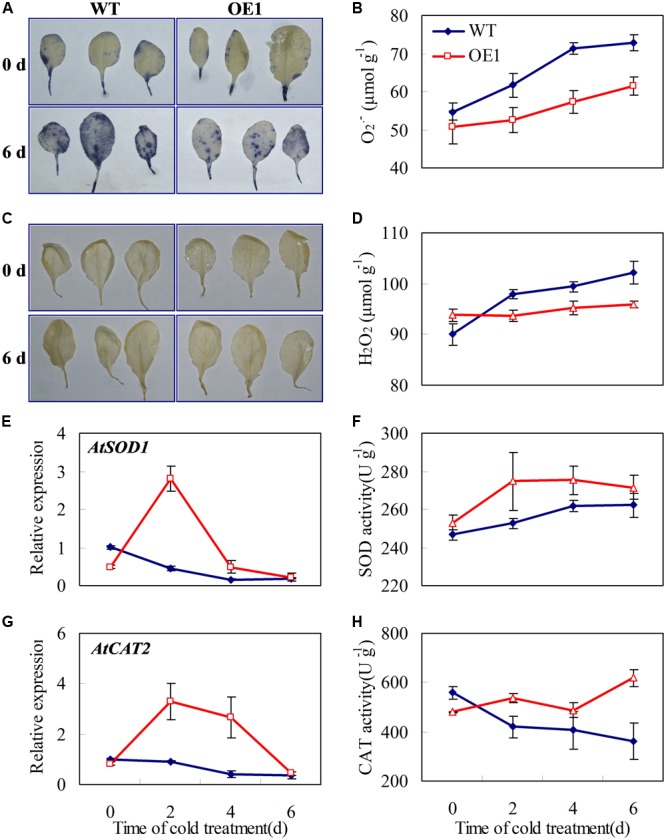
Effects of *CsGR-RBP3* over-expression on O_2_^⋅-^ and H_2_O_2_ accumulations, and CAT and SOD expression in *Arabidopsis*. 22-day-old seedlings were subjected to 0°C for 6 days. **(A)** O_2_^⋅-^ location in *Arabidopsis* leaf was assayed with nitro blue tetrazolium (NBT). **(B)** H_2_O_2_ location was assayed with diaminobenzidine (DAB). Changes of O_2_^⋅-^ **(C)** and H_2_O_2_ **(D)** concentrations in *Arabidopsis* plants under chilling stress. Expression patterns of *AtSOD1* **(E)** and *AtCAT2* **(G)**. The expression data were normalized to 100% (1.0) at 0 day of the wild-type plants. Changes of SOD **(F)** and CAT **(H)** activities. Gene names and the corresponding Genbank accession numbers are: *AtSOD1* (NM_100757.4) and *AtCAT2* (NM_119675.4). Data are presented as means ±*SE* (*n* = 3).

PsCA not only increased *CsGR-RBP3* gene expression (**Figures 2, 3A**) and protein accumulation (Wang et al., 2018), it also activated the antioxidant system in cucumber (Wang and Zhu, 2017). This raises the question of whether *CsGR-RBP3* up-regulation is related to the antioxidant system activation in cucumber. If overexpression of cucumber *CsGR-RBP3* gene could enhance *Arabidopsis* antioxidant enzymes, this would suggest there is a relationship. **Figure 7** showed that under chilling stress conditions, the accumulation of O_2_^⋅-^ and H_2_O_2_ in leaves of transgenic *Arabidopsis* plants overexpressing *CsGR-RBP3* was reduced compared with the wild-type plants (**Figures 7A–D**), whereas the gene expression and enzyme activities of CAT and SOD were significantly enhanced in the transgenic plants (**Figures 7E–H**). As the two transgenic lines are highly consistent in accumulation of O_2_^⋅-^ and H_2_O_2_ and activities of CAT and SOD (Supplementary Figure S6), OE1 was used as a representative for determination of gene expression.

### *CsGR-RBP3* Regulates the Expression of Stress-Related Genes in Transgenic *Arabidopsis* Plants

**FIGURE 8:**
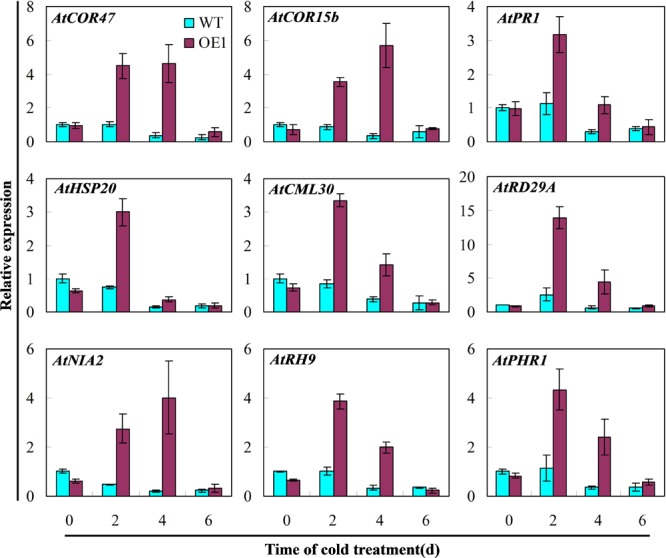
Expression of defense genes involved in different defense pathways in *CsGR-RBP3-*over-expressed *Arabidopsis* plants. 22-day-old plants of the wild-type (WT) and *35S::CsGR-RBP3* plants (OE1) were subjected to 0°C for 6 days. The relative expression of stress-related genes was evaluated by qRT-PCR using gene-specific primers (Supplementary Table S2) and the expression data were normalized to 100% (1.0) at 0 day of the wild-type plants. Gene names and the corresponding Genbank accession numbers are: *AtCOR47* (NM_101894.4), *AtCOR15b* (NM_129814.3), *AtPR1* (NM_127025.3), *AtHSP20* (NM_127488.3), *AtCML30* (NM_127129.2), *AtRD29A* (NM_124610.3), *AtNIA2* (NM_103364.3), *AtRH9* (NM_113129.4), and *AtPHR1* (NM_179320.2). Data are presented as means ±*SE* (*n* = 3).

To determine the impact of overexpression of cucumber *CsGR-RBP3* in *Arabidopsis* on *Arabidopsis* defense systems, expression of nine genes involved in various defense pathways in response to cold stress were analyzed. As shown in **Figure 8**, the expression of *AtCOR47, AtCOR15b, AtPR1, AtHSP20, AtCML30, AtRD29A, AtNIA2, ATRH9*, and *AtPHR1* did not increase in wild-type *Arabidopsis* plants under cold stress. However, all were highly upregulated in *Arabidopsis* plants overexpressing *CsGR-RBP3* after the seedlings were exposed to 0°C for 2 days.

## Discussion

In this study, PsCA treatment produced significantly lower CII, REL, and SDI, and higher ratios of *F*_v_/*F*_m_ than the control (**Figure 1**). As CII, REL, and SDI are regarded as indicators of chilling injury severity (Liu et al., 2016; Wang and Zhu, 2017), these results collectively suggest that PsCA induced strong chilling tolerance in cold-stored cucumber, consistent with former studies (Kashash et al., 2016; Li et al., 2017; Zhang et al., 2017; Wang et al., 2018). Over the years, many studies have shown that *GR-RBP* genes are highly upregulated in response to cold stress, and examples include *AtRZ-1a, GR-RBP4, AtRZ-1b*, and *AtRZ-1c* from *Arabidopsis* (Kim et al., 2005, 2010a; Kwak et al., 2005), *BnGRP1* from *B. napus* (Kim et al., 2012), three GRP2s (Kwak et al., 2013), and *GRP7* from *C. sativa* (Kwak et al., 2016). There are also exceptions, and the accumulation of transcripts and proteins of all the *LeGRP1a-c* genes from harvested tomato fruit did not increase at 4°C (Müller et al., 2014). In the current study, based on the responses to cold stress conditions, the six cucumber *CsGR-RBP*s genes could be divided into two categories, with *CsGR-RBP2* being enhanced, and the other five not upregulatedby cold stress (**Figure 2**). This is the first demonstration that the majority of the *GR-RBP* gene family members from a plant species are not induced by cold stress, but whether this is characteristic of harvested fruit remains to be clarified. *Arabidopsis* is a chilling-tolerant plant (Hasdai et al., 2006) whereas cucumber is chilling-sensitive (Liu et al., 2016). Whether this sensitivity affects the expression pattern of *GR-RBP* genes in response to cold stress remains to be investigated. Therefore, whether and how the cucumber *GR-RBP* genes are involved in cold tolerance induction is worth further investigation. PsCA treatment highly upregulated expression of the five genes that were not enhanced by cold stress (**Figure 2**), suggesting their upregulation could be related to chilling tolerance.

Endogenous ABA and NO play important roles in regulation of stress-related genes and signal transduction (Desikan et al., 2004). The NO-dependent signaling network is closely related to ABA responses (Desikan et al., 2004). Plant *GR-RBP* genes may be regulated by ABA (Aneeta et al., 2002; Shinozuka et al., 2006; Kim et al., 2008a; Wang et al., 2012). It is, therefore, worth investigating how inhibition of endogenous ABA and NO affects chilling tolerance and *CsGR-RBP3* gene expression induced by PsCA. The results here showed that pre-treatment with TS or L-NAME significant increased chilling severity compared with PsCA alone (**Figures 3B–D**). In addition, *CsGR-RBP3* expression was markedly down-regulated by inhibition of endogenous ABA and NO before PsCA treatment (**Figure 3A**), implying *CsGR-RBP3* upregulation induced by PsCA involved biosynthesis of ABA and NO. These results confirm the connection between increased gene expression of *CsGR-RBP3* and enhanced chilling tolerance in harvested cucumber fruit. It is noted that the peak values for CsGR-RBP3 expressions in PsCA-treated fruit in **Figures 2, 3** were not on the same day. Maybe this resulted from the differences in physiological state between batches of cucumber produced in different growth conditions, as **Figures 2, 3** are from different experiment conducted in different seasons. However, this suggest that regardless of producing conditions, the cucumber fruit exposed to PsCA could display induced chilling tolerance and enhanced *CsGR-RBP3* expression.

The defining character of GRPs is the presence of a glycine-rich region containing conserved (GGX)_n_ repeats that form the basis upon which GRPs are divided into five major groups (Lorković, 2009). The protein sequence of CsGR-RBP3 contains the characteristic 13 (GGX)_n_ repeats and an RRM conserved domain (Supplementary Figures S2, S3), and, therefore, CsGR-RBP3 falls into the class IV category of RBPs (**Figure 4A**), indicating CsGR-RBP3 is a typical glycine-rich RNA-binding protein. However, class IV is split into two groups (**Figure 4A**), and CsGR-RBP3 shares identities between 15.1 and 48.2% with GR-RBPs from six other plants (Supplementary Table S3). This suggests GR-RBPs from different plants produce highly diverse protein structures implying that their cellular localizations and functions are also likely to be diverse.

Studies have shown that different GR-RBPs from the same plant can have different sub-cellular localizations. In *Arabidopsis*, AtGR-RBP2 is located in the mitochondria (Vermel et al., 2002), AtGR-RBP7, AtGR-RBP8, and AtRZ-1a are located in the nucleus and cytoplasm (Schöning et al., 2008; Kim et al., 2010a; Kwak et al., 2016), whereas AtRZ-1b and AtRZ-1c proteins are localized in the nucleus (Kim et al., 2010a). In the present study, cucumber CsGR-RBP3 was located in the mitochondria (**Figure 4B** and Supplementary Table S4), implying the gene could play a role in maintaining mitochondrial function under low temperature.

Plants overexpressing *AtRZ-1a* (Kim et al., 2005) and *AtGRP5* (Mangeon et al., 2009) produced longer roots. In the present study, transgenic *Arabidopsis* plants over-expressing *CsGR-RBP3* displayed longer roots, and more and larger leaves compared with wild-type plants (**Figures 5A–D**). This suggests the gene could have breeding potential in raising the productivity of leafy vegetables. Reports on the role of *GRPs* overexpression in relation to chilling or freezing tolerance vary. Some studies show overexpression of *GRPs* enhanced chilling or freezing tolerance (Nomata et al., 2004; Kim et al., 2005, 2008a, 2012; Kim J.Y. et al., 2007; Kwak et al., 2013, 2016), whereas others showed no conference of chilling or freezing tolerance (Kwak et al., 2005; Kim et al., 2010a; Yang et al., 2014). In the present study, two transgenic *Arabidopsis* lines overexpressing *CsGR-RBP3* showed significantly higher leaf growth rate (**Figures 5E,F**), lower EL, and higher *F*_v_/*F*_m_ ratios (**Figures 5G,H**) following exposure to 0°C for 6 days, and higher survival rates after freezing treatment at -20°C (**Figures 6A,B**). These results, together with those that show PsCA treatment significantly enhanced *CsGR-RBP3* gene expression, and that TS+ and L-NAME+PsCA treatments downregulated expression, strongly indicate that *CsGR-RBP3* plays a positive role in cold and freezing stress tolerance in cucumber fruit.

Reactive oxygen species, which include hydrogen peroxide (H_2_O_2_) and superoxide radicals (O_2_^⋅-^), affect many cellular functions by damaging nucleic acids, oxidizing proteins, and stimulating lipid peroxidation (Foyer and Noctor, 2005). Abnormal temperatures can cause a strong oxidative burst that may lead to cellular damage and death (Sharma et al., 2012). The antioxidant enzymes, such as SOD and CAT, are crucial for maintenance of cell integrity (Asada, 2006). Transgenic *Arabidopsis* lines overexpressing *MpGR-RBP1* had reduced ROS accumulation under salt stress (Tan et al., 2014). *CaGR-RBP1* negatively regulated *CaPIK1*-triggered cell death and defense responses by suppressing ROS accumulation in pepper (Kim et al., 2015). CAT and mitochondria-encoded SOD are modulated by *AtGR-RBP2* under cold stress (Kim J.Y. et al., 2007). In the current study, *CsGR-RBP3* overexpression in *Arabidopsis* plants produced lower ROS levels, and higher CAT and SOD gene expression and enzyme activities than the wild type plants, under chilling stress conditions (**Figure 7**). This suggests that enhanced anti-oxidant enzyme activities in the transgenic plants partly contributed to the increased chilling resistance of the transgenic plants. Considering that CsGR-RBP3 is mitochondria-targeted (**Figure 4B** and Supplementary Table S4), and mitochondrial membranes are thought to be the main intracellular source of ROS (Dröge, 2002), plant GR-RBPs appear to exhibit RNA chaperone activity during the cold-adaptation process (Kim J.S. et al., 2007; Kim et al., 2010b). SOD and CAT are significantly enhanced in gene expression and enzyme activity by PsCA treatment (Wang and Zhu, 2017), and the results of the current study suggest CsGR-RBP3 modulated antioxidant enzymes, such as SOD and CAT, in cucumber to regulate generation of ROS.

The upregulation of CAT and SOD, the two major ROS scavengers, could only account for part of the chilling tolerance in transgenic *Arabidopsis*, as the plant defense system consists of different pathways that function in a coordinated manner. To determine whether and how other defense pathways are influenced, expression of 9 other *Arabidopsis* genes, i.e., *AtCOR47, AtCOR15b, AtPR1, AtHSP20, AtCML30, AtRD29A, AtNIA2, AtRH9*, and *AtPHR1* have been investigated in transgenic *Arabidopsis* overexpressing cucumber *CsGR-RBP3*. These genes play roles in cold acclimation, systemic acquired resistance, response to cold stress, ROS, RNA metabolism, and DNA damage repair. The *CBF* (CRT/DRE-binding factor) genes play central roles in plant cold acclimation (Zhao et al., 2016) and are induced by cold (Fowler and Thomashow, 2002). The cold-induced CBF proteins directly bind to the CRT/DRE *cis*-elements in the promoters of downstream cold-regulated (COR) genes and activate their expression (Jiang et al., 2017). Upregulation of *COR* genes increases chilling and freezing tolerance (Kurepin et al., 2013). The *PR1* gene is regarded as a marker for systemic acquired resistance (Carella et al., 2016). PsCA induced accumulation of three PR proteins in cold-stored cucumbers (Wang et al., 2018). The upregulated *PR* genes are usually associated with the enhancement of resistance to pathogens and chilling (Goyal et al., 2016). Heat shock proteins are often associated with plant responses to cold stress and reactive oxygen species (Sun et al., 2002), and HSP20 represents the most abundant of the HSPs in plants (Lopes-Caitar et al., 2013). Calmodulin (CaM) and CaM-like (CML) proteins function in cold acclimation through the regulation of cold-responsive gene expression (Perochon et al., 2011). Repression of CAMTA1, CAMTA2, and CAMTA3 (a CaM-dependent transcriptional activator) genes was recently reported to reduce the cold-induced accumulation of transcripts for CBF1 and CBF2 (Kim et al., 2013). AtCML30 is localized in the mitochondria (Chigri et al., 2012). Cold acclimation induces ABA accumulation in plants (Cuevas et al., 2008) and the *AtRD29A* gene is controlled by ABA (Knight et al., 2004). Nitric reductase (NR)-dependent nitric oxide production is involved in cold acclimation and freezing tolerance in *Arabidopsis* (Zhao et al., 2009). NR is encoded by *AtNIA2*, which accounts for 90% of the total NR activity (Wilkinson and Crawford, 1993). Antioxidant systems are regulated by nitric oxide-mediated post-translational modifications (Begaramorales et al., 2016). RNA helicases (RH) play vital roles in many aspects of RNA metabolism and have been implicated in cold adaptation (Kim et al., 2008b). In *Arabidopsis* plants, cold stress markedly induces upregulation of *AtRH9*, and overexpression of *AtRH9* enhanced freezing tolerance (Kim et al., 2008b). DNA damage repair proteins are important for plant cell survival. The UV-B induced cyclobutane pyrimidine dimer photolyase (PHR) gene is essential for plant survival following exposure to UV-B (Li et al., 2015). Transgenic analysis revealed that overexpression of *AtPHR1* reduced DNA damage in rice (Takahashi et al., 2011). In the present study, over-expression of *CsGR-RBP3* in *Arabidopsis* plants resulted in significant up-regulation of all nine genes, suggesting that CsGR-RBP3 regulated multiple defense pathways.

## Conclusion

Pre-storage cold acclimation at 10°C significantly enhanced chilling tolerance of harvested cucumber fruit stored under chilling stress conditions. Expression of *CsGR-RBP3* displayed little change in responses to chilling stress, but was significantly upregulated following PsCA treatment. Inhibition of endogenous NO and ABA before PsCA treatment clearly downregulated *CsGR-RBP3* expression and significantly aggravated symptoms of chilling injury. *Arabidopsis* plants overexpressing CsGR-RBP3 displayed faster growth at 23°C, stronger chilling tolerance at 0°C, and higher survival rate at -20°C than wild-type plants. The *CsGR-RBP3-*overexpressing *Arabidopsis* lines displayed lower ROS levels and higher CAT and SOD expression and activities than the wild-type plants under cold stress conditions. Nine *Arabidopsis* genes involved in various defense responses were all upregulated in CsGR-RBP3-overexpressed *Arabidopsis* plants. These results strongly suggest that *CsGR-RBP3* plays a positive role in regulating cold and freezing stress tolerance in plants.

## Author Contributions

SZ conceived and oversaw the work. BW and FS performed the experiments. BW made the tables and figures. SZ, BW, and GW wrote the manuscript. All authors read and approved the manuscript.

## Conflict of Interest Statement

The authors declare that the research was conducted in the absence of any commercial or financial relationships that could be construed as a potential conflict of interest.

## Footnotes

1 http://cucurbitgenomics.org/

2 http://web.expasy.org/compute_pi/
