# Supplementary material for: A Glycine-Rich RNA-Binding Protein, CsGR-RBP3, Is Involved in Defense Responses Against Cold Stress in Harvested Cucumber (Cucumis sativus L.) Fruit
Source: Front Plant Sci. 2018 Apr 23;9:540. doi: 10.3389/fpls.2018.00540 (PMC5925850; doi:10.3389/fpls.2018.00540)
Supplement: Supplementary file 1 [file Table_1.DOC]

**Supplementary material**

**Table S1.** **Identification of the differentially accumulated proteins by MS in peels of harvested cucumber exposed 3 d of PsCA treatment plus 9 d of cold storage at 5^o^C in comparison to the 3 d of PsCA treatment.**

| Spot no. | Fold change^a^ | Homologous protein | NCBI accession | Mr(kDa)/pI^b^ | Score^c^ | NP^d^ |
| --- | --- | --- | --- | --- | --- | --- |
| 1(↑) | 3.06 | Transcription factor BTF3 homolog 4-like isoform X2 | gi|449439239 | 17.46/6.62 | 96 | 6 |
| 5(↑) | 3.39 | Membrane-associated 30 kDa protein | gi|778689862 | 36.3/9.21 | 134 | 5 |
| 6(↑) | 3.12 | Biotin carboxyl carrier protein of acetyl-CoA carboxylase | gi|778709585 | 29.66/9.15 | 117 | 6 |
| 4(↑) | 2.57 | Glycine-rich RNA-binding protein blt801 | gi|778656094 | 28.46/5.07 | 168 | 5 |
| 2(↓) | 0.28 | RuBisCO large subunit-binding protein subunit beta | gi|449452644 | 65.03/5.86 | 134 | 6 |
| 3(↓) | 0.31 | RuBisCO large subunit-binding protein subunit beta | gi|449452644 | 65.03/5.86 | 76 | 3 |
| 7(↓) | 0.32 | V-type proton ATPase subunit E-like | gi|449460539 | 26.24/6.47 | 147 | 7 |
| 8(↓) | 0.38 | ATP synthase subunit O | gi|449436299 | 27.65/9.33 | 142 | 8 |

^a^ Average fold change of a protein abundance between 3 d of PsCA + 9d of cold storage versus the 3 d of PsCA treatment.

^b^ The mass (kDa) and *pI* of identified proteins.

^c^ Protein scores reported after searching against the Cucumber Genomic database. Protein scores greater than 61 are significant (p<0.05).

^d^ The number of matched peptides.
