# Supplementary material for: A Glycine-Rich RNA-Binding Protein, CsGR-RBP3, Is Involved in Defense Responses Against Cold Stress in Harvested Cucumber (Cucumis sativus L.) Fruit
Source: Front Plant Sci. 2018 Apr 23;9:540. doi: 10.3389/fpls.2018.00540 (PMC5925850; doi:10.3389/fpls.2018.00540)
Supplement: Supplementary file 2 [file Table_2.DOC]

**Supplementary material**

**Table S2. Specific primers used in PCR amplification.**

| Application | Primer sequences (5’–3’) | Amplicon | Accession no. | Restriction Site |
| --- | --- | --- | --- | --- |
| Full length cloning for *CsGR-RBP3* | F1: GGGGTACCATGCAATTATTCCCCACACG | 507 bp | XM_004137389.2 | *Kpn I* |
|  | F2: GGACTAGTTCAGTTTTTGTCACCACCACC |  |  | *Spe I* |
| Subcellular localization for CsGR-RBP3 | F3: GGGGTACCATGCAATTATTCCCCACACG | 504 bp | XM_004137389.2 | *Kpn I* |
|  | F4: GGACTAGTGTTTTTGTCACCACCACCATAG |  |  | *Spe I* |
| *CsActin* | F: AGGCCGTTCTGTCCCTCTAC | 150 bp | AB698859 |  |
|  | R: CAGTAAGGTCACGACCAGCA |  |  |  |
| *AtActin* | F: GCCAGTGGGCATTGGATTTG | 108 bp | NM_112046.4 |  |
|  | R: CTCCAATCATGTGGTTCGGC |  |  |  |
| qPCR analysis for *CsGR-RBP-RZ1A* | F: GGTTCTTTGCGGCTGTCTTG | 75 bp | XM_011660470.1 |  |
|  | R: ATCCCACCATGTAAGCTGGC |  |  |  |
| qPCR analysis for *CsGR-RBP2* | F: ATGAGAAGCTCCGAGAAGCC | 74 bp | XM_011656066.1 |  |
|  | R: CGTCGCCCTATCGGTTACAA |  |  |  |
| qPCR analysis for *CsGR-RBP3* | F: GCAGCCTTCCAAGCTGTCTA | 119 bp | XM_004137389.2 |  |
|  | R: ACTTGCTGAACGCTACCCTC |  |  |  |
| qPCR analysis for *CsGR-RBP4* | F:GAGGATTTGCCTTCCTCCGT  R: TCACCCTGCCATCCAGAAAC | 90 bp | XM_011651346.1 |  |
| qPCR analysis for *CsGR-RBP5* | CCTTGCTTGGGCCACTGATA | 111 bp | XM_004148797.2 |  |
|  | AATCCTCTCGACCTTCCGGT |  |  |  |
| qPCR analysis for *CsGR-RBP-blt801* | F: GGGCAATTATGGCGGAGGAA | 73 bp | XM_011650146.1 |  |
|  | R: ACCAACACTTCCACGGTTGT |  |  |  |
| qPCR analysis for *AtCAT2* | F: CAAGTGTGGGGTGTCGAGAG | 117 bp | [NM_119675.4](https://www.ncbi.nlm.nih.gov/entrez/viewer.fcgi?db=nucleotide&id=1063726906) |  |
|  | R: ACCCCAAAATGTCAAAACCAAGTT |  |  |  |
| qPCR analysis for *AtSOD1* | F: TCCTGAGATCACAAAGGCCAA | 101 bp | [NM_100757.4](https://www.ncbi.nlm.nih.gov/entrez/viewer.fcgi?db=nucleotide&id=1063681730) |  |
|  | R: TCGCCTTCCTGGGTGAAAAA |  |  |  |
| qPCR analysis for *AtCOR47* | F: GACGTGTCTAATGGCCCACA | 96 bp | [NM_101894.4](https://www.ncbi.nlm.nih.gov/entrez/viewer.fcgi?db=nucleotide&id=1063685074) |  |
|  | R: TAAAAGAGGTGGGAACGGGC |  |  |  |
| qPCR analysis for *AtCOR15b* | F: CAACTTGATGGCCGACCTCT | 84 bp | [NM_129814.3](https://www.ncbi.nlm.nih.gov/entrez/viewer.fcgi?db=nucleotide&id=1063703093) |  |
|  | R: AGAAGAGTTTTCGTTGGTTCGT |  |  |  |
| qPCR analysis for *AtPR1* | F: CTCGGAGCTACGCAGAACAA | 87 bp | [NM_127025.3](https://www.ncbi.nlm.nih.gov/entrez/viewer.fcgi?db=nucleotide&id=1063700386) |  |
|  | R: CGCTACCCCAGGCTAAGTTT |  |  |  |
| qPCR analysis for *AtHSP20* | F: CGTGGATGGAGGACGAGTTT | 119 bp | [NM_127488.3](https://www.ncbi.nlm.nih.gov/entrez/viewer.fcgi?db=nucleotide&id=1063700758) |  |
|  | R: ATCGCCGGTGATTTCCACAA |  |  |  |
| qPCR analysis for *AtCML30* | F: TTTGAACGGCGATGGGAAGA | 96 bp | [NM_127129.2](https://www.ncbi.nlm.nih.gov/entrez/viewer.fcgi?db=nucleotide&id=145359988) |  |
|  | R: ACCATCCTGTTGCAGTCCTC |  |  |  |
| qPCR analysis for *AtRD29A* | F: ACGTTTGCTCCAAGTGGTGA | 79 bp | [NM_124610.3](https://www.ncbi.nlm.nih.gov/entrez/viewer.fcgi?db=nucleotide&id=1063740776) |  |
|  | R: CCTCCAACGTTATCGGGGTC |  |  |  |
| qPCR analysis for *AtNIA2* | F: CATTTTCCTTTGCGCCACCA | 90 bp | [NM_103364.3](https://www.ncbi.nlm.nih.gov/entrez/viewer.fcgi?db=nucleotide&id=1063688319) |  |
|  | R: AGCTCGAAGTAGCCAACCAC |  |  |  |
| qPCR analysis for *AtRH9* | F: GCCGTTACTCTGGTGGTTCA | 126 bp | [NM_113129.4](https://www.ncbi.nlm.nih.gov/entrez/viewer.fcgi?db=nucleotide&id=1063713105) |  |
|  | R: CAAACCCACCAAAGCTGCTC |  |  |  |
| qPCR analysis for *AtPHR1* | F: TTGCCTTCCCTCCATTTTTCG | 101 bp | [NM_179320.2](https://www.ncbi.nlm.nih.gov/entrez/viewer.fcgi?db=nucleotide&id=1063682817) |  |
|  | R: CTAATGTGCGCCGGTTATGC |  |  |  |
