# Supplementary material for: A Glycine-Rich RNA-Binding Protein, CsGR-RBP3, Is Involved in Defense Responses Against Cold Stress in Harvested Cucumber (Cucumis sativus L.) Fruit
Source: Front Plant Sci. 2018 Apr 23;9:540. doi: 10.3389/fpls.2018.00540 (PMC5925850; doi:10.3389/fpls.2018.00540)
Supplement: Supplementary file 3 [file Table_3.DOCX]

**Supplementary material**

**Table S3. Comparison of cucumber GR-RBP3 (CsGR-RBP3) with GR-RBPs from five other plant species. Values indicate percentage identity, which were obtained by EMBOSS Stretcher (https://www.ebi.ac.uk/Tools/psa/emboss_stretcher/). See Figure 4 for accession no.**

|  | 1 | 2 | 3 | 4 | 5 | 6 | 7 | 8 | 9 | 10 | 11 | 12 | 13 | 14 | 15 | 16 | 17 | 18 | 19 | 20 | 21 |
| --- | --- | --- | --- | --- | --- | --- | --- | --- | --- | --- | --- | --- | --- | --- | --- | --- | --- | --- | --- | --- | --- |
| 1:CsGR-RBP3 | 100 |  |  |  |  |  |  |  |  |  |  |  |  |  |  |  |  |  |  |  |  |
| 2:CmGR-RBP3 | 48.2 | 100 |  |  |  |  |  |  |  |  |  |  |  |  |  |  |  |  |  |  |  |
| 3:AtGR-RBP2 | 31.1 | 50.6 | 100 |  |  |  |  |  |  |  |  |  |  |  |  |  |  |  |  |  |  |
| 4:AtGR-RBP3 | 20.9 | 34.3 | 29.8 | 100 |  |  |  |  |  |  |  |  |  |  |  |  |  |  |  |  |  |
| 5:AtGR-RBP4 | 36.2 | 42.9 | 62.0 | 22.3 | 100 |  |  |  |  |  |  |  |  |  |  |  |  |  |  |  |  |
| 6:AtGR-RBP5 | 24.2 | 39.2 | 31.0 | 53.3 | 26.0 | 100 |  |  |  |  |  |  |  |  |  |  |  |  |  |  |  |
| 7:AtGR-RBP6 | 29.1 | 44.8 | 43.6 | 24.7 | 43.0 | 27.6 | 100 |  |  |  |  |  |  |  |  |  |  |  |  |  |  |
| 8:AtGR-RBP7 | 21.0 | 37.6 | 41.9 | 32.8 | 31.0 | 34.7 | 31.4 | 100 |  |  |  |  |  |  |  |  |  |  |  |  |  |
| 9:AtGR-RBP8 | 17.3 | 38.5 | 43.5 | 30.8 | 30.6 | 31.4 | 34.0 | 77.3 | 100 |  |  |  |  |  |  |  |  |  |  |  |  |
| 10:NtGR-RBP1 | 21,7 | 42.6 | 43.5 | 31.1 | 31.7 | 33.8 | 34.2 | 71.9 | 74.0 | 100 |  |  |  |  |  |  |  |  |  |  |  |
| 11:NtGR-RBP2 | 36.9 | 46.3 | 60.9 | 23.6 | 61.6 | 26.8 | 38.1 | 31.6 | 31.6 | 33.9 | 100 |  |  |  |  |  |  |  |  |  |  |
| 12:NtGR-RBP3 | 25.1 | 37.9 | 31.0 | 47.0 | 29.2 | 41.7 | 28.6 | 33.2 | 30.7 | 31.0 | 29.6 | 100 |  |  |  |  |  |  |  |  |  |
| 13:NtGR-RBP4 | 24,9 | 39.0 | 34.7 | 41.7 | 32.0 | 43.5 | 27.5 | 34.7 | 30.7 | 31.7 | 32.4 | 62.1 | 100 |  |  |  |  |  |  |  |  |
| 14:SlGR-RBP1 | 22,6 | 38.8 | 39.7 | 33.0 | 29.4 | 37.9 | 31.2 | 70.1 | 70.6 | 78.9 | 29.5 | 34.3 | 35.9 | 100 |  |  |  |  |  |  |  |
| 15:SlGR-RBP2 | 26.0 | 40.3 | 35.8 | 45.7 | 31.1 | 45.0 | 28.7 | 34.7 | 31.4 | 35.4 | 30.7 | 48.7 | 50.2 | 38.0 | 100 |  |  |  |  |  |  |
| 16:SlGR-RBP3 | 26.8 | 29.6 | 32.3 | 17.2 | 36.0 | 20.7 | 30.3 | 21.1 | 21.7 | 22.5 | 32.6 | 22.4 | 22.8 | 22.5 | 21.8 | 100 |  |  |  |  |  |
| 17:SlGR-RBP4 | 26.2 | 40.5 | 34.5 | 42.1 | 29.0 | 41.5 | 29.0 | 31.3 | 29.8 | 31.0 | 31.9 | 59.4 | 68.7 | 31.7 | 45.3 | 24.6 | 100 |  |  |  |  |
| 18:OsGR-RBP2 | 21.9 | 40.3 | 42.3 | 31.7 | 34.4 | 35.2 | 35.3 | 61.8 | 69.8 | 74.6 | 33.9 | 30.3 | 31.7 | 72.8 | 34.6 | 22.7 | 32.5 | 100 |  |  |  |
| 19:OsGR-RBP3 | 15.1 | 20.2 | 17.9 | 27.2 | 14.6 | 25.2 | 16.6 | 24.4 | 23.2 | 22.7 | 14.6 | 21.9 | 23.3 | 23.9 | 23.3 | 12.8 | 22.6 | 21.7 | 100 |  |  |
| 20:OsGR-RBP4 | 26.3 | 29.0 | 29.6 | 15.5 | 32.6 | 15.8 | 28.5 | 19.6 | 19.1 | 18.7 | 33.3 | 16.6 | 19.3 | 19.0 | 19.5 | 36.2 | 18.3 | 21.1 | 10.1 | 100 |  |
| 21:OsGR-RBP10 | 18.8 | 31.3 | 28.6 | 47.3 | 23.4 | 42.1 | 22.0 | 30.9 | 28.3 | 29.0 | 24.9 | 42.8 | 40.1 | 32.8 | 37.2 | 18.9 | 37.9 | 31.2 | 21.6 | 18.0 | 100 |
