# Supplementary material for: A Glycine-Rich RNA-Binding Protein, CsGR-RBP3, Is Involved in Defense Responses Against Cold Stress in Harvested Cucumber (Cucumis sativus L.) Fruit
Source: Front Plant Sci. 2018 Apr 23;9:540. doi: 10.3389/fpls.2018.00540 (PMC5925850; doi:10.3389/fpls.2018.00540)
Supplement: Supplementary file 4 [file Table_4.DOC]

**Supplementary material**

**Table S4. The sub-cellular localization of CsGR-RBP3 predicted by softberry (http://www.softberry.com/).**

| Location weights | LocDB | PotLocDB | Neural Nets | Pentamers | Integral |
| --- | --- | --- | --- | --- | --- |
| Nuclear | 0.0 | 0.0 | 0.44 | 0.00 | 0.00 |
| Plasma membrane | 0.0 | 0.0 | 0.03 | 0.46 | 0.11 |
| Extracellular | 0.0 | 0.0 | 0.00 | 0.57 | 0.01 |
| Cytoplasmic | 0.0 | 0.0 | 3.19 | 0.03 | 0.96 |
| Mitochondrial | 10.0 | 0.0 | 0.00 | 1.07 | 8.67 |
| Endoplasm. retic. | 0.0 | 0.0 | 0.06 | 0.15 | 0.00 |
| Peroxisomal | 0.0 | 0.0 | 0.00 | 0.31 | 0.00 |
| Lysosomal | 0.0 | 0.0 | 0.00 | 0.09 | 0.00 |
| Golgi | 0.0 | 0.0 | 0.02 | 0.38 | 0.03 |
| Vacuolar | 0.0 | 0.0 | 0.00 | 0.04 | 0.22 |
