# Supplementary figures and images for: A Glycine-Rich RNA-Binding Protein, CsGR-RBP3, Is Involved in Defense Responses Against Cold Stress in Harvested Cucumber (Cucumis sativus L.) Fruit
Source: Front Plant Sci. 2018 Apr 23;9:540. doi: 10.3389/fpls.2018.00540 (PMC5925850; doi:10.3389/fpls.2018.00540)

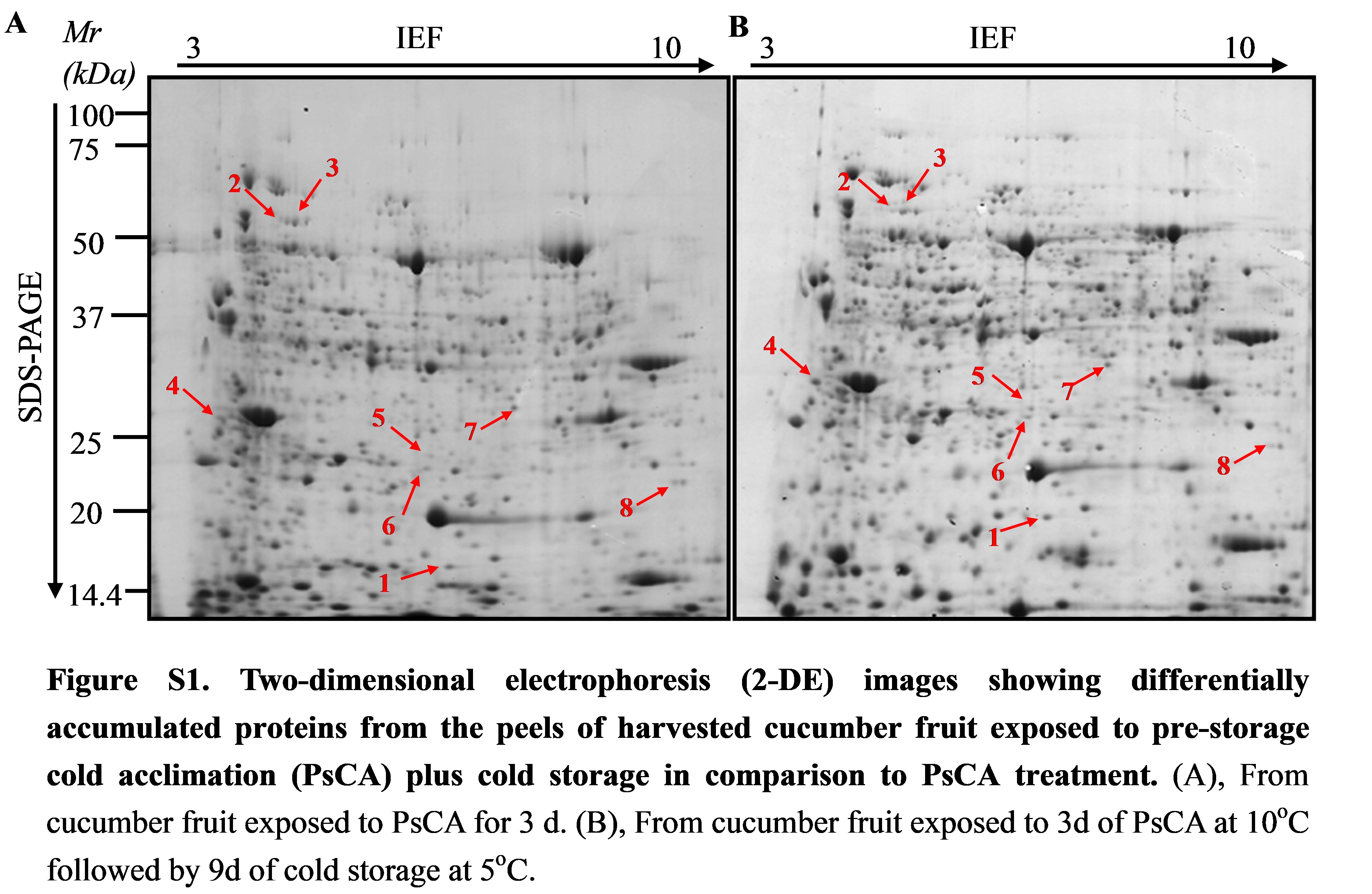

Supplement: Supplementary file 5 [file Image_1.JPEG]

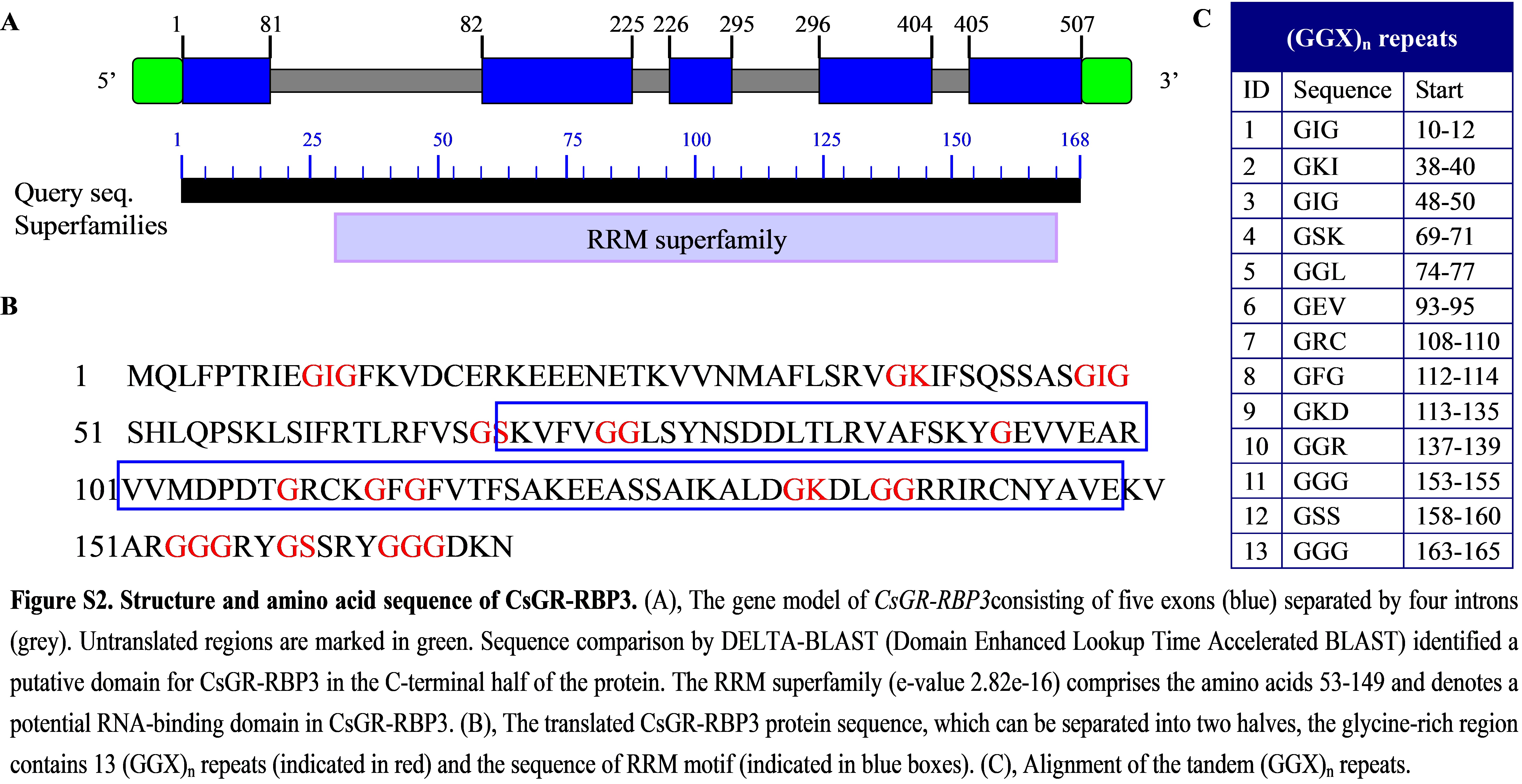

Supplement: Supplementary file 6 [file Image_2.JPEG]

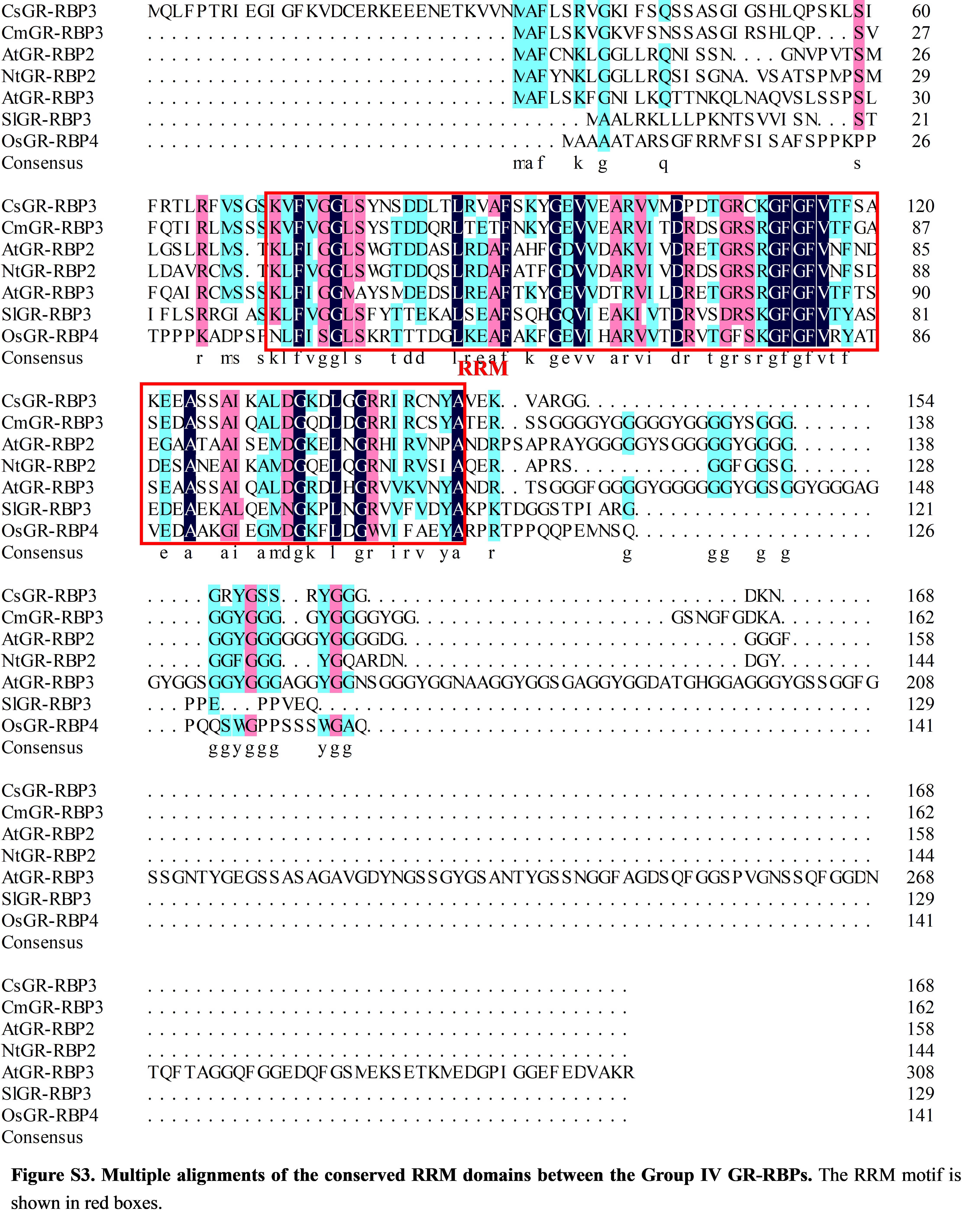

Supplement: Supplementary file 7 [file Image_3.JPEG]

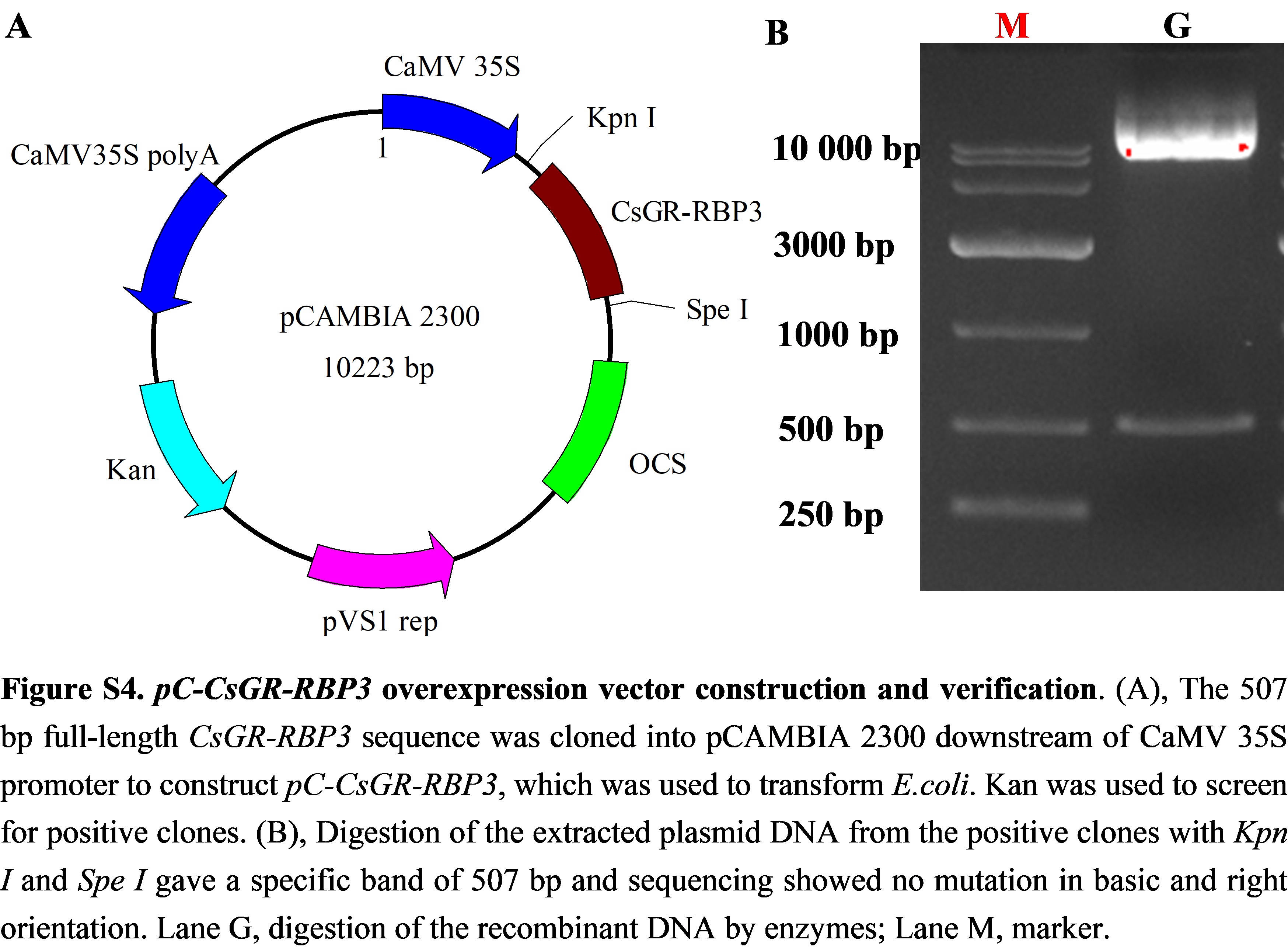

Supplement: Supplementary file 8 [file Image_4.JPEG]

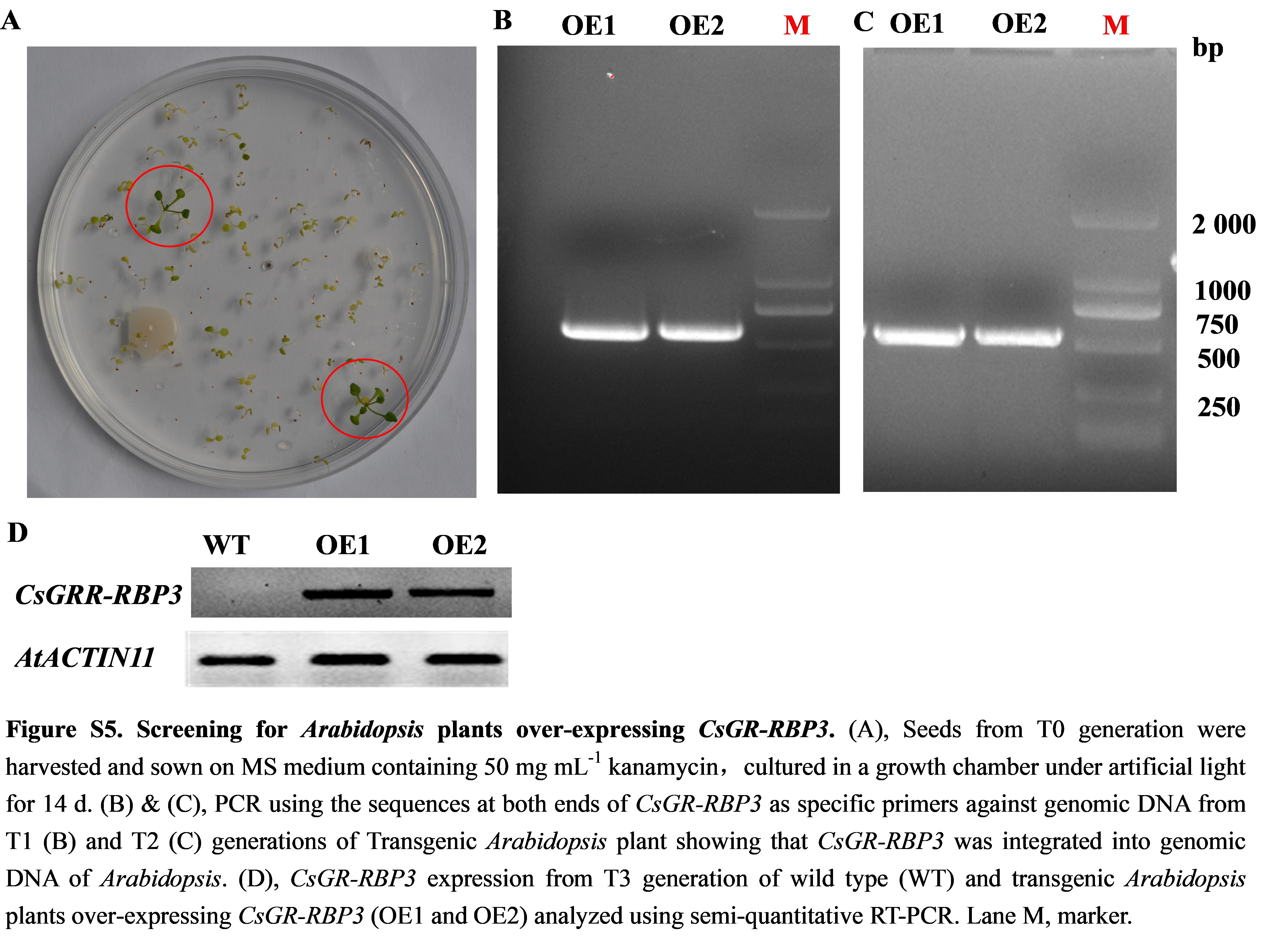

Supplement: Supplementary file 9 [file Image_5.JPEG]

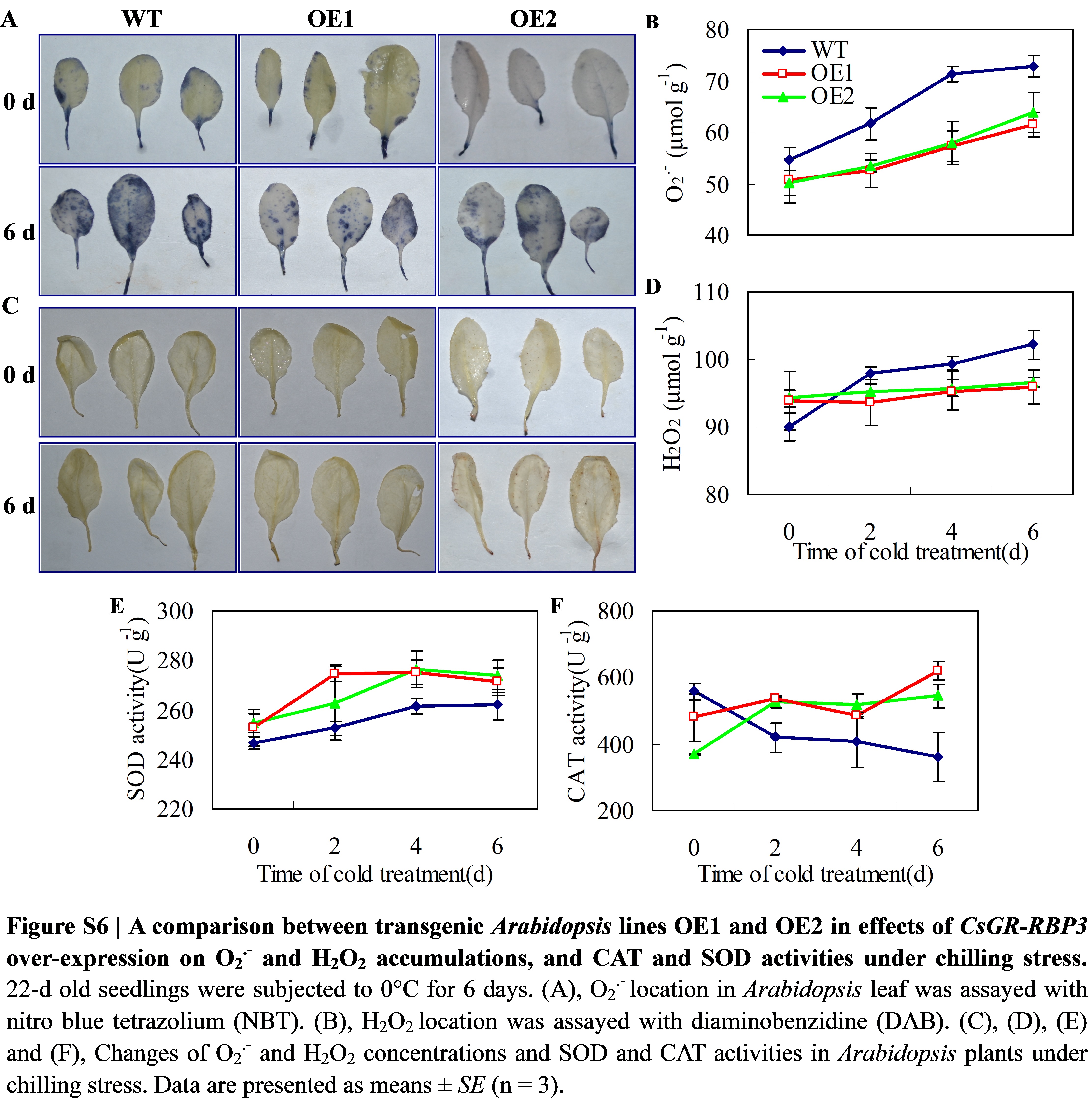

Supplement: Supplementary file 10 [file Image_6.JPEG]
